# A Study on the Incidence of Hand or Wrist Injuries in CrossFit Athletes

**DOI:** 10.7759/cureus.13818

**Published:** 2021-03-11

**Authors:** Amr Tawfik, Brian M Katt, Francis Sirch, Michael E Simon, Fortunato Padua, Daniel Fletcher, Pedro Beredjiklian, Michael Nakashian

**Affiliations:** 1 Division of Hand Surgery, Rothman Orthopedic Institute, Philadelphia, USA; 2 Department of Orthopedic Surgery, Rutgers Robert Wood Johnson Medical School, New Brunswick, USA; 3 Department of Orthopedic Surgery, Mercy Health, Toledo, USA

**Keywords:** hand, wrist, crossfit, injury

## Abstract

Purpose

The purpose of this study was to investigate the reported rates and characteristics of injuries among CrossFit athletes with specific attention to the hand and wrist. We further sought to identify trends and associations of these injuries by examining demographic data.

Methods

A questionnaire was created to capture self-reported information on the incidence of hand or wrist injuries and their associations in CrossFit athletes. It was distributed between April 2020 and July 2020 to athletes training at CrossFit affiliated gyms in the New York and Pennsylvania regions. Bivariate logistic regression analysis was used to identify factors correlated with an injury.

Results

A total of 270 responses (97.5% response rate) were available for final analysis. The median age of respondents was 34 years and 72.2% had been participating in CrossFit for at least two years. CrossFit athletes reported injury rates of 62.2% while engaged in CrossFit training and 20.4% reported an injury specific to the hand or wrist. The majority of hand or wrist injuries occurred after one year of CrossFit training (65.4%). The majority of reported initial injuries occurred to the wrist (75.4%); subsequently, 29.1% reported reinjuring the same region. While 58.2% reported not yet having healed or taking longer than one month to feel fully healed, 72.8% reported returning to unmodified training within one month. Only 15 respondents reported seeking medical attention from a physician for their injury. Physicians generally recommended a training break of over one month, but only two patients reported taking a break this long. Male sex and length of participation in CrossFit were associated with an increased risk of developing a hand or wrist injury.

Discussion

Hand and wrist injuries represent a significant proportion of injuries among CrossFit athletes. CrossFit practitioners are potentially returning to unmodified training too early following injury, leaving them susceptible to further injury. Physicians and therapists must consider these findings and address both therapies and preventative measures for these types of injuries with their patients.

## Introduction

CrossFit is a high-intensity strength and conditioning exercise program in which calisthenics, Olympic weightlifting, and traditional weightlifting exercises are completed in rapid succession and in a limited time interval. CrossFit exercises are grouped into “workouts of the day” that are completed individually or in groups and provide the scalability necessary to be inclusive of a diverse range of athletic abilities. CrossFit was initially founded in the early 2000s and has since become a worldwide phenomenon. CrossFit’s popularity is likely associated with its perceived health benefits. This belief stems from existing literature supporting improvements in lean body mass, improved performance, and fat mass reduction in age-matched cohorts following high intensity, single-modal exercises including CrossFit training [1,2].

As CrossFit has grown in popularity, there has been g­­­rowing concern about an increased risk of injury in its participants. While a 2010 study by the Consortium for Health and Military Performance and the American College of Sports Medicine corroborated extreme conditioning programs’ benefits, they also noted the increased risk of musculoskeletal injury and possible rhabdomyolysis due to complex movements, lack of adequate rest intervals, and high training volume [3]. These factors could lead to early fatigue, oxidative stress, and unsafe movement completion [3,4]. Further studies have attempted to clarify the risks and types of injuries associated with CrossFit training. They found that the risk of injury within a year of CrossFit training typically ranged between 20% and 30% [5-8]. Of note, shoulder injuries were often found to be one of the most common injuries in CrossFit athletes [8-10].

However, there is little in the literature regarding CrossFit-related injuries specific to the hand or wrist. The wrist is unique compared to most other joints, as it lacks support from local muscles, instead of relying on support from the tendons and local ligaments [11]. Many CrossFit movements require repetitive hyperextension of the hand and wrist, making it necessary to understand the epidemiology for these types of injuries [12]. For this reason, our study aims to investigate the reported injury rates and characteristics among CrossFit athletes, with specific attention to the hand and wrist. We further sought to identify trends and associations for these injuries by examining demographic data.

## Materials and methods

This study was an epidemiological study based on a questionnaire administered to CrossFit participants training at CrossFit affiliated gyms in the New York and Pennsylvania regions. Between April 2020 and July 2020, we coordinated with owners of these gyms to distribute the questionnaire electronically to their gym members. 

The questionnaire consisted of three sections with instructions. The first section consisted of demographic data, including age, sex, smoking status, length of time involved in CrossFit, number of days participating in a week, and participation in CrossFit competitions. Participation in CrossFit competitions was defined as participating in a CrossFit sanctioned competition at least once a year. Additionally, participants were asked if they had sustained any CrossFit-related injury and if they had sustained a CrossFit-related injury specifically to the hand or wrist. A CrossFit related injury was defined as any of the following which occurred as the result of CrossFit training: (1) inability to train for greater than one week; (2) needing to modify training duration, activity, or intensity for greater than two weeks; (3) any complaint that led to a doctor visit.

The second section was available only to those who had reported sustaining a CrossFit-related injury to the hand or wrist. It consisted of questions categorizing the type and number of injuries. The survey included the length of time participating in CrossFit before the injury, number of independent injuries, the specific area that was injured, if they had been reinjured in the same area, and if they had worn wrist wraps before the injury. Additionally, athletes were asked about the time until they returned to unmodified training, how long it took until they felt 100% healed, and if they had seen a doctor for the injury.

The third and final section was available only to those who had reported seeing a doctor for their injury. It included questions on the length of time between the injury and the physician appointment, the injury diagnosis, the length of training break recommended, the length of the training break taken, and if the injury required surgical intervention.

Age was reported as median and interquartile range. All other responses to the questionnaire were reported as count and percentage of total responses. Bivariate logistic regression was performed for the reported demographic data to assess their effect on injury rates specific to the hand or wrist. Results from the regression analysis were reported as odds ratios with a 95% confidence interval. A p-value of 0.05 was selected as the significance level for these tests.

## Results

A total of 277 questionnaires were collected throughout the study duration. Seven questionnaires were excluded from the final analysis due to incomplete data. The final analysis included a total of 270 questionnaires. 

The median age of athletes was 34 years, consisting of 50.9% males and 49.1% females. Of those surveyed, 79.6% reported having never smoked. The majority of those surveyed reported having participated in CrossFit for at least two years (72.2%), with 60% reporting that they participated in CrossFit four to five days a week on average. Participation in one CrossFit sanctioned competition annually was reported 41.9% while 62.2% reported having sustained an injury while CrossFit and 20.4% reported an injury specific to the hand or wrist (Table 1).

**Table 1:** Participant Demographics The age was reported as median and interquartile range. All other data are reported as count and percent of the total population. IQR: interquartile range

Participant Demographics	
Variable	N = 270
Age, median (IQR)	34.0 (29.0-40.0)
Sex	
Male	137 (50.9)
Female	132 (49.1)
Have you ever smoked?	
Yes	55 (20.4)
No	215 (79.6)
How long have you done CrossFit?	
0-6 months	3 (1.1)
6-12 months	26 (9.6)
1-2 years	46 (17.0)
2-5 years	104 (38.5)
>5 years	91 (33.7)
On average, how many days per week do you participate in CrossFit?	
1 to 3	52 (19.3)
4 to 5	162 (60.0)
6 to 7	56 (20.7)
Do you participate in CrossFit competitions?	
Yes	113 (41.9)
No	157 (58.1)
Have you ever sustained an injury while CrossFit training?	
Yes	168 (62.2)
No	102 (37.8)
Have you ever sustained an injury while CrossFit training to the hand or wrist?	
Yes	55 (20.4)
No	214 (79.6)

Of the 55 respondents that reported an injury to the hand or wrist, 65.4% reported that it had been over a year since their first injury. Most of them have suffered only one injury to this region (67.3%), with 21.8% having two independent injuries to the hand or wrist. CrossFit-related injuries in this region occurred in the wrist 76.4% of the time. The reinjury rate in this group was 29.1%. Wrist wraps were employed 34.5% of the time prior to their injury. While 58.2% report not yet healing or taking longer than one month to feel 100% healed, 72.8% report returning to unmodified training within a month. Only 27.3% report seeing a doctor for the injury (Table 2).

**Table 2:** Injury Rates and Characteristics of the Hand or Wrist for CrossFit Athletes All data reported as count and percent of the total population.

Injury Rates and Characteristics of the Hand or Wrist for CrossFit Athletes	
Variable, n (%)	N = 55
Length of time before first injury	
0-30 days	3 (5.5)
1-3 months	2 (3.6)
3-6 months	5 (9.1)
6-12 months	9 (16.4)
1-2 years	17 (30.9)
2+ years	19 (34.5)
How many independent injuries did you have to the hand or wrist?	
1	37 (67.3)
2	12 (21.8)
3	2 (3.6)
4+	4 (7.3)
What part of the hand/wrist was injured?	
Finger	8 (14.5)
Hand	5 (9.1)
Wrist	42 (76.4)
Have you re-injured the same hand/wrist?	
Yes	16 (29.1)
No	39 (70.9)
Did you wear wrist wraps for lifting prior to injury?	
Yes	19 (34.5)
No	36 (65.5)
How long did it take until you returned to unmodified training?	
< 1 week	15 (27.3)
1-2 weeks	10 (18.2)
2-4 weeks	15 (27.3)
4-12 weeks	6 (10.9)
12+ weeks	9 (16.4)
How long did it take until you felt 100% healed?	
< 1 week	2 (3.6)
1-2 weeks	11 (20.0)
2-4 weeks	10 (18.2)
4-12 weeks	11 (20.0)
12+ weeks	15 (27.3)
Not yet healed	6 (10.9)
Did you see a doctor for the injury?	
Yes	15 (27.3)
No	40 (72.7)

Only 15 patients visited a physician for their CrossFit-related injury, reportedly seeing the physician in less than two weeks from the injury’s time, 86.7% of the time. Three were diagnosed with a fracture, and six were diagnosed with a sprain or ligament rupture. The doctors most frequently recommended a training suspension of four weeks or greater, but only two participants reported taking a break this long. Only one patient reported requiring surgical intervention for their injury (Table 3). 

**Table 3:** CrossFit Athletes That Visited a Physician All data reported as count and percent of the total population.

CrossFit Athletes That Visited a Physician	
Variable, n (%)	N = 15
How long from your injury did you wait to see a physician?	
<1 week	7 (46.7)
1-2 weeks	6 (40.0)
2-4 weeks	2 (13.3)
What were you diagnosed with?	
Fracture	3 (20.0)
Sprain or ligament rupture	6 (40.0)
Other	6 (40.0)
How long of a training break were you recommended to take?	
<1 week	4 (26.7)
1-2 weeks	2 (13.3)
2-4 weeks	3 (20.0)
4-12 weeks	6 (40.0)
How long of a break did you actually take from training?	
<1 week	5 (33.3)
1-2 weeks	3 (20.0)
2-4 weeks	5 (33.3)
4-12 weeks	2 (13.3)
Did you require surgery for the injury?	
Yes	1 (6.7)
No	14 (93.3)

Male sex was associated with an increased risk of having an injury to the hand or wrist, with an odds ratio of 2.10 (p = 0.0160). Length of CrossFit participation for greater than five years was associated with a 2.75 fold risk of developing a hand or wrist injury (p = 0.001). No other demographic factors were associated with increased risk (Table 4).

**Table 4:** Factors Associated With Increased Risk of Hand/Wrist Injury *p-Value ≤ 0.05 was considered to be significant. CI: confidence interval

Factors Associated With Increased Risk of Hand/Wrist Injury			
Variable	Odds ratio	CI	p-Value
Sex (male)	2.10	1.13-3.89	0.016*
Age	1.00	0.964-1.04	0.960
Competition	1.59	0.875-2.88	0.129
Smoking	0.84	0.393-1.79	0.652
Length of participation	2.75	1.50-5.05	0.001*
Days per week	1.07	0.670-1.71	0.777

## Discussion

This study aimed to investigate the risks for injury among CrossFit athletes and the characteristics of those injuries, with a particular interest in the hand and wrist. We found that 62.2% of CrossFit athletes reported sustaining an injury at least once during CrossFit training and that 20.4% of CrossFit athletes had injured their hand or wrist at least once. Further, we found that males and those participating in CrossFit for over five years were significantly more likely to injure their hands or wrists.

The currently available literature on CrossFit-related injuries is highly variable, suggesting injury rates ranging between 12.8% and 73.5% [5,9,10,13]. This variability is due to the heterogeneity both in the periods over which injuries were tracked and in the definitions of what constitutes an injury. Adjusting for the duration of training hours leads to an injury rate of 0.27-3.3 per 1000 training hours in CrossFit [10]. This is comparable to the reported injury rates for Olympic weightlifting, rugby, and gymnastics and significantly lower than the injury rates reported for American football, ice hockey, and soccer [14-19].

CrossFit’s most commonly injured areas are typically cited as the shoulder, back, and knees, with rates of 22.6-39.0%, 12.9-36.0%, and 14.1-16.1%, respectively [5,9,20,21]. The high rate of injury in these anatomic regions appears to be related to two main factors. Firstly, the Olympic-style lifts utilized in CrossFit often place these regions, particularly the shoulder, at the extremes of their range of motion [14]. Secondly, CrossFit’s highly repetitive nature leads to significant muscle fatigue, potentially resulting in a loss of proper form [6,9]. Our study found a 20.4% injury rate to the hand or wrist for CrossFit athletes, comparable to these other highly injured regions. CrossFit movements, such as power cleans, front squats, and handstand push-ups place the wrist beyond its physiologic range of motion in rapid succession [12]. It is not unsurprising that 76.4% of reported injuries between the fingertip and the distal forearm were reported to have occurred in the wrist.

It is essential to note that most hand and wrist injuries did not occur until after at least one year of CrossFit participation. When they did occur, it was not uncommon for the same area to be reinjured. Reinjury is expected, as it has been shown that previous injuries carry a four-fold risk of future injury [22]. Furthermore, 32.7% of respondents reporting a hand or wrist injury noted having more than one independent injury to the hand or wrist. This is partly explained by the fact that a majority of participants returned to unmodified training within one month, despite a significant percentage of them not having fully recovering in that time. Participants who visited a physician for a hand or wrist injury were likely to take a shorter training break than recommended. Athletes returning to unmodified activity before adequate recovery have increased re-injury risks due to the involved tissues or bones being weaker and subsequent decreases in muscle strength and range of motion [23]. For this reason, physicians need to better explain the risks of returning to unmodified training early and offer alternative exercises until patients can safely return to CrossFit training. Furthermore, other avenues of injury prevention, such as wrist wraps, should be examined in future studies, as they were not used in a significant proportion of those who developed hand or wrist injuries. 

When we evaluated the relationships between demographic factors and the risk of hand or wrist injury, only the length of CrossFit participation and male sex were associated with an increased risk of injury. Since our study evaluated all injuries related to CrossFit training, it seems intuitive that as people participate in CrossFit for a more extended period of time, there were more opportunities to sustain a hand or wrist injury. It is not entirely clear why males are more likely to injure themselves than females. However, Weisenthal et al. had found that females were significantly more likely to seek out trainers or coaches for CrossFit guidance and training instruction than males [6]. They further found a significant correlation between the use of coach supervision and a lower injury rate. This suggests that the increased incidence of injury in males in our study may be due to either unsupervised training or improper form. Age was not associated with an increased risk of injury, suggesting that CrossFit may be safely implemented for all ages represented in our study. Surprisingly, the number of days per week training was not associated with an increased risk of hand or wrist injury. Both Feito et al. and Minghelli and Vicente had found that training less than three times per week was associated with an increased risk of injury [5,7]. This may be related to the increases in muscle strength and flexibility and the improvements in the technique that are associated with increased frequency of training.

We are aware of the limitations associated with our study design. There is an issue of sampling bias, as the surveys were distributed at CrossFit-affiliated gyms. For this reason, it is possible that those who were currently injured and not training were inadvertently excluded from our study. Furthermore, our questionnaire was distributed at CrossFit-affiliated gyms in the New York and Philadelphia regions, which may not be broadly applicable to all CrossFit practitioners. Additionally, there was the potential for recall bias, as some of the questions required participants to recall information regarding their injuries. Finally, the specific data captured regarding hand or wrist injuries were limited to the 55 participants reporting those injuries, impacting the strength of the findings for these particular questions.

## Conclusions

In conclusion, hand and wrist injuries represent a significant proportion of injuries that occur in CrossFit athletes. CrossFit practitioners are potentially returning to unmodified training too early following injury, leaving them susceptible to further injury. Physicians and therapists need to consider these findings and address therapies and preventative measures for these types of injuries with patients.

## References

[1] Cavedon V, Milanese C, Marchi A, Zancanaro C (2020). Different amount of training affects body composition and performance in high-intensity functional training participants. PLoS One.

[2] Giessing J, Eichmann B, Steele J, Fisher J (2016). A comparison of low volume “high-intensity-training” and high volume traditional resistance training methods on muscular performance, body composition, and subjective assessments of training. Biol Sport.

[3] Bergeron MF, Nindl BC, Deuster PA (2011). Consortium for Health and Military Performance and American College of Sports Medicine consensus paper on extreme conditioning programs in military personnel. Curr Sports Med Rep.

[4] Elkin JL, Kammerman JS, Kunselman AR, Gallo RA (2019). Likelihood of injury and medical care between CrossFit and traditional weightlifting participants. Orthop J Sport Med.

[5] Feito Y, Burrows EK, Tabb LP (2018). A 4-year analysis of the incidence of injuries among CrossFit-trained participants. Orthop J Sport Med.

[6] Weisenthal BM, Beck CA, Maloney MD, DeHaven KE, Giordano BD (2014). Injury rate and patterns among CrossFit athletes. Orthop J Sport Med.

[7] Minghelli B, Vicente P (2019). Musculoskeletal injuries in Portuguese CrossFit practitioners. J Sports Med Phys Fitness.

[8] Sprey JWC, Ferreira T, de Lima M V, Duarte AJ, Jorge PB, Santili C (2016). An epidemiological profile of CrossFit athletes in Brazil. Orthop J Sport Med.

[9] Hak PT, Hodzovic E, Hickey B (2013). The nature and prevalence of injury during CrossFit training. [Epub ahead of print]. J Strength Cond Res.

[10] Gardiner B, Devereux G, Beato M (2020). Injury risk and injury incidence rates in CrossFit. J Sports Med Phys Fitness.

[11] Kijima Y, Viegas SF (2009). Wrist anatomy and biomechanics. J Hand Surg Am.

[12] Vella M (2018). New Anatomy for Strength & Fitness Training: An Illustrated Guide to Your Muscles in Action Including Exercises Used in CrossFit®, P90X®, and Other Popular Fitness Programs.

[13] Klimek C, Ashbeck C, Brook AJ, Durall C (2018). Are injuries more common with CrossFit training than other forms of exercise?. J Sport Rehabil.

[14] Calhoon G, Fry AC (1999). Injury rates and profiles of elite competitive weightlifters. J Athl Train.

[15] Kolt GS, Kirkby RJ (1999). Epidemiology of injury in elite and subelite female gymnasts: a comparison of retrospective and prospective findings. Br J Sports Med.

[16] Rechel JA, Yard EE, Comstock RD (2008). An epidemiologic comparison of high school sports injuries sustained in practice and competition. J Athl Train.

[17] Lorentzon R, Wedrèn H, Pietilä T (1988). Incidence, nature, and causes of ice hockey injuries. A three-year prospective study of a Swedish elite ice hockey team. Am J Sports Med.

[18] DeLee JC, Farney WC (1992). Incidence of injury in Texas high school football. Am J Sports Med.

[19] Williams S, Trewartha G, Kemp S, Stokes K (2013). A meta-analysis of injuries in senior men’s professional Rugby Union. Sports Med.

[20] Montalvo AM, Shaefer H, Rodriguez B, Li T, Epnere K, Myer GD (2017). Retrospective injury epidemiology and risk factors for injury in CrossFit. J Sports Sci Med.

[21] Lima PO, Souza MB, Sampaio TV, Almeida GP, Oliveira RR (2020). Epidemiology and associated factors for CrossFit-related musculoskeletal injuries: a cross-sectional study. J Sports Med Phys Fitness.

[22] Fuller CW, Bahr R, Dick RW, Meeuwisse WH (2007). A framework for recording recurrences, reinjuries, and exacerbations in injury surveillance. Clin J Sport Med.

[23] Creighton DW, Shrier I, Shultz R, Meeuwisse WH, Matheson GO (2010). Return-to-play in sport: a decision-based model. Clin J Sport Med.

